# The role of Evi/Wntless in exporting Wnt proteins

**DOI:** 10.1242/dev.201352

**Published:** 2023-02-13

**Authors:** Lucie Wolf, Michael Boutros

**Affiliations:** German Cancer Research Center (DKFZ), Division of Signalling and Functional Genomics and Heidelberg University, BioQuant and Department of Cell and Molecular Biology, 69120 Heidelberg, Germany

**Keywords:** Wnt signalling, Wnt secretion, Development, Cancer, Evi, Wntless/Wls, PORCN/Porcupine

## Abstract

Intercellular communication by Wnt proteins governs many essential processes during development, tissue homeostasis and disease in all metazoans. Many context-dependent effects are initiated in the Wnt-producing cells and depend on the export of lipidated Wnt proteins. Although much focus has been on understanding intracellular Wnt signal transduction, the cellular machinery responsible for Wnt secretion became better understood only recently. After lipid modification by the acyl-transferase Porcupine, Wnt proteins bind their dedicated cargo protein Evi/Wntless for transport and secretion. Evi/Wntless and Porcupine are conserved transmembrane proteins, and their 3D structures were recently determined. In this Review, we summarise studies and structural data highlighting how Wnts are transported from the ER to the plasma membrane, and the role of SNX3-retromer during the recycling of its cargo receptor Evi/Wntless. We also describe the regulation of Wnt export through a post-translational mechanism and review the importance of Wnt secretion for organ development and cancer, and as a future biomarker.

## Teaser

**Summary:** This Review summarises mechanisms of Wnt ligand export and recent structural insights, with a focus on the Wnt transport protein Evi/Wntless.

## Introduction

Multicellular life depends on patterning decisions and coordinated tissue growth during development and homeostasis in adults – processes that are controlled by intricate cell-cell communication. This exchange of information requires the coordinated action of proteins over short and long ranges, and at the organismal level. Wnt signalling pathways are essential signal transduction routes that orchestrate many context-dependent cellular decisions. Their activities are closely intertwined with other signalling cascades, such as epidermal growth factor receptor (EGFR), transforming growth factor β (TGFβ) and Hedgehog signalling (Albrecht et al., 2021; Mittermeier and Virshup, 2022; Rim et al., 2022).

Wnt ligands are secreted cysteine-rich glycoproteins of around 40 kDa, which carry (with few exceptions) a lipid modification. Wnt proteins can be grouped in 13 subfamilies with high evolutionary conservation in animals, but are absent from unicellular organisms, plants and fungi (Holstein, 2012). During development, Wnt ligands regulate proliferation, cell fate decisions and cell migration, and can act as morphogens to control, for example, target gene transcription in a concentration-dependent manner (Stapornwongkul and Vincent, 2021; Steinhart and Angers, 2018). In adults, Wnt ligands play essential roles in tissue homeostasis, turnover and repair by regulating adult stem cell populations. Moreover, mutations in components of the Wnt signalling pathways can lead to diseases, such as cancer (Mittermeier and Virshup, 2022; Parsons et al., 2021; Rim et al., 2022; Steinhart and Angers, 2018; Zhan et al., 2017).

To our knowledge, all branches of the Wnt signalling network rely on the secretion of Wnt ligands from Wnt-producing cells. Wnt export is tightly regulated and mediated by a dedicated export machinery that is dependent on two conserved proteins: Porcupine (PORCN) and Evi (also known as Wntless/Wls, Sprinter, GPR177 and MIG-14). After the co-translational import into the endoplasmic reticulum (ER), Wnt proteins are lipid modified by Porcupine. Lipid modification is necessary for their interaction with the cargo protein Evi/Wls and their anterograde transport to the extracellular space (Bänziger et al., 2006; Bartscherer et al., 2006; Goodman et al., 2006; Siegfried et al., 1994; Takada et al., 2006; Willert et al., 2003). During trafficking through several cellular compartments, Evi/Wls interacts with multiple molecular machines and has been used as ‘model substrate’ to study diverse processes, such as endoplasmic reticulum (ER)-associated degradation or retromer function during endosomal trafficking (Glaeser et al., 2018; McGough et al., 2018; Wolf et al., 2021). Not surprisingly, deregulated Wnt secretion can result in diseases that are present at birth or can lead to malignant transformation of cells and the development of cancers later in life (Chai et al., 2021; Grzeschik et al., 2007; Parsons et al., 2021; Wang et al., 2007). Therefore, Wnt export in Wnt-dependent diseases has become a target for drug development (reviewed by Jung and Park, 2020; Zhang and Lum, 2018).

The topography, trafficking and regulation of Evi/Wls have been extensively studied since its discovery in 2006 (Bänziger et al., 2006; Bartscherer et al., 2006; Goodman et al., 2006). Fifteen years later, advancements in cryogenic electron microscopy (cryo-EM) allowed the study of the structure of Evi/Wls in complex with Wnt at a molecular resolution (Nygaard et al., 2021; Zhong et al., 2021). Moreover, structural insights into Wnt acylation by Porcupine provided further insights into Wnt export mechanisms and a basis for future drug development strategies (Liu et al., 2022). Concurrent novel insights into the regulation of Evi/Wls through post-translational modifications, as well as an associated congenital syndrome, make this an appropriate time to review our knowledge on Evi/Wls, Porcupine and Wnt export (Chai et al., 2021; Glaeser et al., 2018; Wolf et al., 2021).

In this Review, we provide an overview of Wnt secretion mechanisms with a focus on the role of Evi/Wls as a dedicated Wnt cargo protein and its Wnt-dependent regulation, as well as its role in development and disease. We discuss cell culture and animal models used to study Wnt secretion and conclude by highlighting unanswered questions and topics to be explored further. Unfortunately, owing to space restrictions, we do not extensively discuss mechanisms of intercellular Wnt transfer, which were recently excellently reviewed (Mittermeier and Virshup, 2022; Routledge and Scholpp, 2019).

## Wnts are glycosylated and lipid modified in the ER

**Fig. 1.**
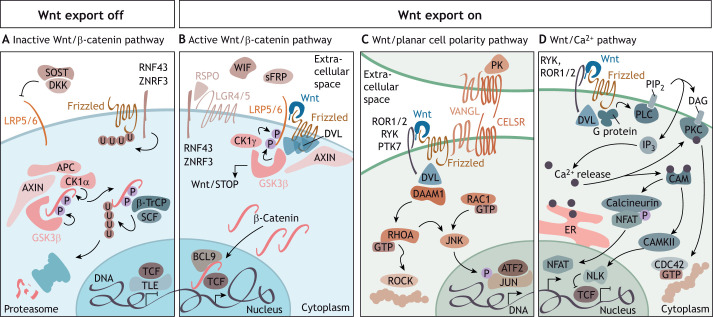
**Wnt signalling pathways.** The Wnt signalling pathways can be divided into β-catenin dependent (‘canonical’) and independent (‘non-canonical’), the most prominent of which are the Wnt/planar cell polarity (PCP) and the Wnt/Ca^2+^ pathways. The β-catenin-dependent pathway is the best studied Wnt signalling branch that activates cell proliferation and differentiation; for example, via the proto-oncogene MYC. β-Catenin is a bifunctional protein involved in cellular adhesion at the plasma membrane and signal transduction in the cytoplasm and the nucleus. The β-catenin-independent pathways are diverse and not extensively studied, mainly due to the lack of well-defined molecular endpoints that are easy to analyse and to pronounced context-dependent effects. They regulate embryonic pattern formation or cell migration, among others. There is considerable overlap between the Wnt pathways, as, for example, the ‘non-canonical’ pathways often inhibit canonical signalling and all Wnt pathways compete for shared proteins, such as Dishevelled (DVL). (A) In the absence of Wnt ligands, β-catenin is phosphorylated by the destruction complex centred on the scaffolding protein AXIN, adenomatous polyposis coli (APC), and the two kinases glycogen synthase kinase 3 (GSK3β) and casein kinase (CK) 1α, allowing its subsequent ubiquitylation by β-transducin repeat-containing protein (β-TrCP) and proteasomal degradation. In the absence of β-catenin, β-catenin-responsive elements in the DNA are bound by transcriptional repressors (TLE). Further regulators of Wnt signalling are the secreted molecules sclerostin (SOST), Dickkopf-related protein (DKK), and the membrane-bound E3 ubiquitin ligases RING finger protein (RNF) 43 and zinc and RING finger 3 (ZNRF3), which target Frizzled receptors for degradation. (B) RNF43 and ZNRF3 can be inhibited by R-Spondin (RSPO), which binds them and leucine-rich repeat-containing G-protein-coupled receptor 4/5 (LGR4/5) to stabilize Frizzled receptors. Further context-dependent regulators of Wnt signalling are Wnt inhibitory factor (WIF) and secreted frizzled-related protein (sFRP). The engagement of ‘canonical’ Wnt ligands, such as human WNT1, WNT3/3A or WNT10A/B, with Frizzled proteins and low-density lipoprotein receptor-related protein 5/6 (LRP5/6) results in the phosphorylation of LRP5/6, e.g. by CK1γ, and in the recruitment of DVL and the destruction complex to the plasma membrane, leading to their inactivation. Stabilized β-catenin translocates into the nucleus to bind T-cell factor (TCF)/lymphoid enhancer factor (LEF) transcription factors and others [e.g. B-cell CLL/lymphoma 9 protein (BCL9)] to activate target gene transcription. Inhibition of the kinase GSK3β also affects many other proteins, an effect that is referred to ‘Wnt/STOP’. (C) The core PCP components [Frizzled, Vang-like protein (VANGL), cadherin EGF LAG seven-pass G-type receptor (CELSR), Prickle-like protein (PK) and DVL] establish an asymmetric network of protein complexes across the membranes of neighbouring cells by inhibiting each other within the same cell and stabilizing each other in bordering cells. Signalling by WNT5A or WNT11, Frizzled and receptor tyrosine kinase-like orphan receptor 1/2 (ROR1/2), receptor-like tyrosine kinase (RYK) or protein tyrosine kinase 7 (PTK7) leads to recruitment and activation of DVL and DVL-associated activator of morphogenesis 1 (DAAM1), and others, and several members of the Ras homolog family (especially RHOA and RAC1), resulting in ROCK-dependent rearrangements of the cytoskeleton or activation of Jun N-terminal kinase (JNK) and transcriptional regulation, e.g. by cAMP-dependent transcription factor 2 (ATF2) and JUN phosphorylation. This is essential to break symmetry during embryonic development and to pattern functional complex organ structures. (D) Wnt/Ca^2+^ signalling is initiated by the interaction of Wnt with Frizzled and RYK or ROR1/2, which results in the intracellular activation of a G protein and cleavage of phosphatidylinositol-4,5-bisphosphate (PIP_2_) by phospholipase C (PLC) into inositol 1,4,5-trisphosphate (IP_3_) and diacylglycerol (DAG). Binding of IP_3_ with its receptor at the ER membrane triggers the release of Ca^2+^ from the endoplasmic reticulum (ER) and activation of the Ca^2+^-dependent enzymes protein kinase C (PKC) and calmodulin (CAM), as well as downstream effectors calcineurin and Ca^2+^/CAM-dependent kinase II (CAMKII), and leads to actin rearrangements by CDC42 and the activation of transcriptional programs via nuclear factor of activated T-cells (NFAT) and nemo-like kinase (NLK), an inhibitor of TCF (reviewed by Acebron and Niehrs, 2016; Albrecht et al., 2021; Humphries and Mlodzik, 2018; Niehrs, 2012; Rim et al., 2022; Söderholm and Cantù, 2021; Zhan et al., 2017).

Wnt ligands are expressed in various tissues throughout organism development and follow a tight spatiotemporal regulation. At the molecular level, Wnts induce different tissue and context-dependent effects that depend on (1) the secreted Wnt ligand, (2) the receptors and co-receptors expressed by the Wnt-receiving cells, and (3) the interplay with other signalling pathways. The engagement of receptors by Wnts on the cell surface triggers several intracellular signal transduction cascades, which are most frequently grouped into β-catenin-dependent (also referred to as ‘canonical’) or -independent (‘non-canonical’) pathways (Fig. 1) (Humphries and Mlodzik, 2018; Niehrs, 2012; Rim et al., 2022). Nevertheless, the upstream signals leading to Wnt expression in the Wnt-secreting cell remain incompletely understood and are probably tissue and context dependent (Inverso et al., 2021; Nusse and Clevers, 2017).

Wnts are co-translationally imported into the ER. Their correct folding and post-translational processing are aided by the ER-resident chaperone, binding immunoglobulin protein (BiP) (Kitajewski et al., 1992; Verras et al., 2008), and they are N-glycosylated at conserved asparagines (Asn) (Smolich et al., 1993). The attachment of glycans to proteins within the secretory routes by the oligosaccharyltransferase complex is essential for protein quality control and protein trafficking (Harada et al., 2019). The functional importance of glycosylation for Wnt signalling was discovered very early after the initial characterization of the first mammalian Wnt protein, mouse Wnt1 (then called int-1; Mason et al., 1992; Papkoff et al., 1987). Biochemical and mutational studies of several canonical and non-canonical Wnt ligands revealed that Wnts could be glycosylated at multiple positions with varying effects on stability, secretion and signalling capacity (Yamamoto et al., 2013). For example, a glycosylation-deficient mutant form of the canonical human WNT3A was expressed more than the wild-type variant and associated with Evi/Wls in a cell culture model but was not secreted efficiently, and the induction of downstream signalling in the Wnt-receiving cell was impaired (Coombs et al., 2010; Komekado et al., 2007). Similarly, non-canonical WNT5A, which had all glycosylation sites removed, was not secreted from HEK293T cells and the capacity of deglycosylated WNT5A to suppress canonical Wnt signalling was reduced (Kurayoshi et al., 2007). Interestingly, human WNT8A harbours a unique glycosylation site (Asn103) that is not conserved in other human WNTs (Nygaard et al., 2021). The glycan attached to this site lies at the interface between Evi/Wls and WNT8A, and its mutation reduced WNT8A expression and signalling capacity in HEK293T cells but its secretion was not affected (Nygaard et al., 2021). On the other hand, the N-glycosylation of *Drosophila* Wingless (Wg, human WNT1 orthologue) was not necessary for active secretion and signalling *in vivo* in the third-instar larval wing disc, a widely used model for Wnt signalling in the fruit fly (Tang et al., 2012). The regulation of Wnts through post-translational modifications was recently reviewed by Yu and Virshup (2022).

## Porcupine – an O-palmitoleoyltransferase

After glycosylation, Wnts are lipid modified (Coombs et al., 2010; Komekado et al., 2007) with palmitoleic acid, a 16-carbon monounsaturated fatty acid (C16:1), on a conserved serine (Ser) by the ER-resident O-palmitoleoyltransferase Porcupine. The involvement of Porcupine in Wnt signalling was first discovered in the early 1990s in *Drosophila* because its genetic loss resulted in a similar embryonic mutant phenotype to the loss of Wg (Siegfried et al., 1994; van den Heuvel et al., 1993). In the years that followed, the importance of Porcupine in Wnt-producing cells was further analysed in various animals (Caricasole et al., 2002; Kadowaki et al., 1996; Tanaka et al., 2000), and the first connections to human cancer were identified (Chen et al., 2008). The enzymatic reaction catalysed by Porcupine and the modified Wnt residues have been extensively studied after the initial observation by Willert and colleagues that Wnts are lipid modified (Gao et al., 2014; Rios-Esteves et al., 2014; Torres et al., 2019; Willert et al., 2003). Biochemical assays (Lee et al., 2019) and structural analysis have confirmed the modification of human WNT3A at Ser209 (Zhong et al., 2021), WNT3 at Ser212 (Hirai et al., 2019) and WNT8A at Ser186 (Nygaard et al., 2021) with palmitoleate. Importantly, the Wnts analysed to date (except for WntD, which has no lipid modification; Ching et al., 2008) have been shown to be mono-palmitoleoylated.

Porcupine belongs to the family of membrane-bound O-acyltransferases (MBOAT), members of which also lipid modify Hedgehog and Ghrelin. However, apart from Wnts, no other target of the catalytic activity of Porcupine has been discovered (Galli et al., 2021; Yu et al., 2021b). Despite the substantial interest in the molecular structure of Porcupine to better understand its catalytic mechanism and how its inhibitors function, structural insights had remained confined to comparisons with other MBOATs until recently (Galli et al., 2021; Yu et al., 2021b). Then, Liu and colleagues developed a novel antibody to aid cryo-EM particle alignment in order to determine the structure of Porcupine (Liu et al., 2022). This study reported four structures of human Porcupine [2.9-3.1 Å resolution, Protein data bank identifiers (PDB IDs) 7URA, 7URC, 7URD and 7URE], including complexes with its substrate palmitoleoyl-CoA or its inhibitor LGK974 (Liu et al., 2022). By building the structure of human Porcupine isoform B, residues 4-222, 234-414 and 425-456, they revealed that Porcupine has 11 transmembrane domains and two opposing cavities in the lipid bilayer that are essential for Wnt palmitoleoylation: the cytosolic cavity can accommodate palmitoleoyl-CoA; and the luminal cavity can be occupied by the Wnt hairpin 2, which is supported by hydrophilic interactions with Porcupine (Liu et al., 2022). The resulting proximity between donor acyl-CoA and Wnt allowed Liu et al. to propose a one-step catalytic mechanism for direct transfer of the lipid; however, a two-step mechanism involving the formation of a lipid-Porcupine intermediate cannot yet be excluded (Liu et al., 2022). The structural model of Porcupine and its inhibitor LGK974 indicates that LGK974 prevents this reaction by blocking the acyl-CoA access site (Liu et al., 2022). Despite these insights, how the transfer of acylated Wnt from Porcupine to Evi/Wls occurs remains to be determined. Structural comparisons indicate that conformational changes and positional adjustments of the Wnt ligand within the lipid bilayer would be necessary to allow lateral transfer in a Porcupine-Evi/Wls-Wnt complex (Liu et al., 2022; Nygaard et al., 2021; Yu et al., 2021b; Zhong et al., 2021).

Besides its important role in Wnt acylation, Porcupine has functions that are independent of its catalytic activity. Porcupine regulates cancer cell proliferation by an unknown but Wnt-independent mechanism (Covey et al., 2012) and functions as a chaperone to stabilize and assemble AMPA-type glutamate receptors in the ER (Erlenhardt et al., 2016; Wei et al., 2020). Porcupine is a highly conserved ER-resident protein; thus, it is conceivable that it has adopted even more general functions throughout evolution; e.g. as a ‘triaging protein’ during ER-associated degradation (ERAD) (Glaeser et al., 2018).

The palmitoleoyl-CoA used by Porcupine for lipidation can be provided exogenously or by stearoyl-CoA desaturase (SCD), a protein that catalyses the formation of mono-unsaturated fatty acids (Rios-Esteves and Resh, 2013). Whereas SCD can produce C16:1 and C18:1 fatty acyl-CoAs, Wnts are preferentially modified with C16:1. This selectivity is mediated by Porcupine itself, which selects palmitoleate through the acyl chain length and the position of its double bond, which leads to a ‘kink’ in its molecular structure (Lee et al., 2019; Liu et al., 2022; Tuladhar et al., 2019), whereas other proteins involved in Wnt secretion and reception (i.e. Evi/Wls and Frizzled) seem to be more flexible with regards to the acyl chain length (Nile et al., 2017; Nygaard et al., 2021). SCD expression is regulated by insulin and sterol-regulatory-element binding protein (SREBP) (Cabrae et al., 2020; Wang et al., 2020), as well as by miRNA-600 and let-7c (El Helou et al., 2017; Zhou et al., 2019). Wnt/β-catenin signalling induces SREBP-dependent transcription of SCD, leading to a positive-feedback loop that regulates lipid homeostasis and can promote liver fibrosis and cancer (Bagchi et al., 2020; Lai et al., 2017). SCD is involved in the anti-tumour T-cell response of various cancer types (Katoh et al., 2022). The possibility of targeting Wnt signalling pathways through the regulation of SCD with small molecules is now being explored (Hosseini et al., 2021; Yu et al., 2021a).

## Evi/Wntless: a dedicated Wnt cargo protein

Only after acylation by Porcupine can Wnts bind to Evi/Wls, an interaction that is necessary for the secretion of most Wnt ligands. Several groups reported the discovery of Evi/Wls in 2006 as a dedicated Wnt transport protein using genetic screening methods in *Drosophila*, and first established the dependency of Wnt secretion on Evi/Wls (Bänziger et al., 2006; Bartscherer et al., 2006; Goodman et al., 2006). The *C. elegans* orthologue of Evi/Wls, MIG-14, was associated with Wnt signalling in the Wnt-producing cell in even earlier studies, but without elucidating the functional connection (Harris et al., 1996; Thorpe et al., 1997). To date, only a few studies have found Wnt-independent functions of Evi/Wls, such as the regulation of neural circuit formation in *C. elegans* and *Drosophila* (Liao et al., 2018) or defects in ER homeostasis and immunity in mouse Evi/Wls knockout dendritic cells (Wang et al., 2021b).

Evi/Wls is highly conserved in metazoans (Holstein, 2012). Curiously only one *EVI/WLS* gene with three transcript variants exists in the human genome, in contrast to 19 WNT or 10 Frizzled genes. The differential regulation and function of these isoforms remain to be thoroughly analysed, but there is evidence that the isoforms differ in their intracellular trafficking capacity and protein interactome (Petko et al., 2019; Yu et al., 2014). The best-studied example is the primate Evi/Wls splice variant 2 (termed WlsX), which differs in its C terminus from canonical isoform 1 and cannot sustain Wnt secretion. As the WlsX and Evi/Wls protein interactome differs slightly, WlsX could have Wnt-independent functions or act as a regulator of Evi/Wls (Petko et al., 2019). Human isoform 1 has a predicted mass of 63 kDa, and the detected size of EVI/WLS in western blots lies between 40 and 50 kDa, often with two visible bands (Wolf et al., 2021; Glaeser et al., 2018; Yu et al., 2014; Coombs et al., 2010). However, the molecular nature of the differences is not yet understood. Biochemical analysis of vertebrate Evi/Wls indicates that the protein is N-glycosylated, but the modified residues or dynamics of this modification remain to be determined (Jin et al., 2010b; Yu et al., 2010). Notably, two structural models of Evi/Wls do not show post-translational modifications. However, to obtain these cryo-EM structures, the protein was produced in human suspension cells lacking N-acetylglucosaminyltransferase I (GnTI) and these cells cannot form complex N-glycans (Nygaard et al., 2021; Zhong et al., 2021).

### Evi/Wls structure determines its function as Wnt cargo protein

Various versions of the molecular structure of Evi/Wls have been proposed, and models ranging from four to eight transmembrane domains have been discussed (Bänziger et al., 2006; Bartscherer et al., 2006; Goodman et al., 2006). In 2021, 15 years after the discovery of Evi/Wls, two cryo-EM structures of human Evi/Wls showed its interaction with WNT8A (3.2 Å resolution, PDB ID 7KC4, Nygaard et al., 2021) or WNT3A (2.2 Å resolution, PDB ID 7DRT, Zhong et al., 2021). The resulting structural models cover amino acids 4-496 (Nygaard et al., 2021) and 3-498 (Zhong et al., 2021) of Evi/Wls (reviewed by Mani et al., 2022). The Evi/Wls C terminus (after the last transmembrane helix) is flexible and could not be solved in these models.

**Fig. 2.**
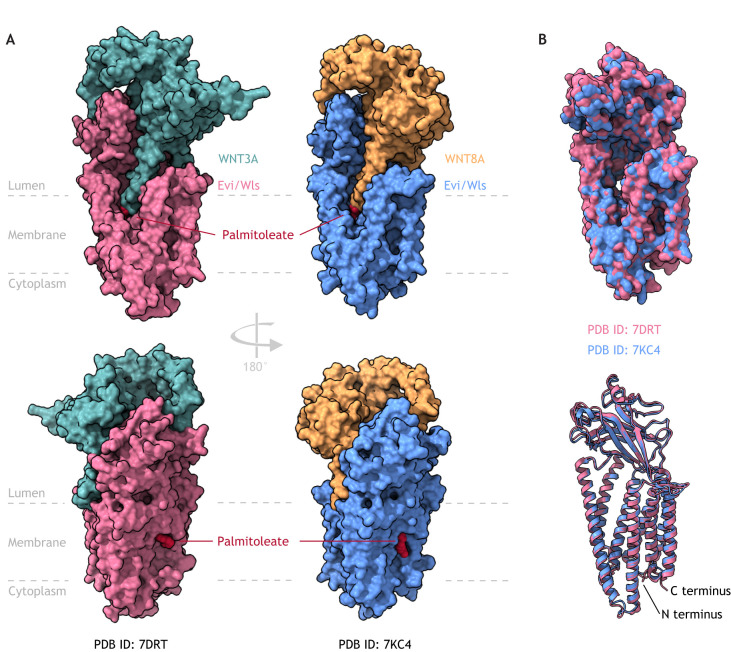
**The Evi/Wntless (Wls) structure determines its function as a Wnt cargo protein.** (A) The structure of human Evi/Wls (pink) in complex with human WNT3A [green; protein data bank identifier (PDB) ID, 7DRT] and human Evi/Wls (blue) and WNT8A (orange; PDB ID 7KC4) reveals the large and complex interaction surface between Evi/Wls and its cargo proteins. Wnts can only bind Evi/Wls when modified with palmitoleate. This long unsaturated fatty acid in the Evi/Wls-Wnt complex is buried in the Evi/Wls transmembrane domain. It is conceivable that lipid-modified Wnts are transferred to Evi/Wls from the acyl-transferase Porcupine through the large lateral opening in the transmembrane domain of Evi/Wls, which leads to the large cavity visible in B. (B) Overlay of the two Evi/Wls structures [Evi/Wls-WNT3A (pink, PDB ID 7DRT) and Evi/Wls-WNT8A (blue, PDB ID 7KC4)] shows that they are very similar, overall, with eight membrane-spanning helices and a luminal or extracellular domain consisting of eight β-strands. Structural figures were generated using UCSF ChimeraX (Version 1.2).

The two models are very similar and reveal that Evi/Wls contains an integral membrane domain composed of eight transmembrane helices and a luminal (or extracellular) domain, which is important for Wnt binding (Fig. 2). Both its N and C termini face the cytoplasm. The transmembrane domains 2-8 share structural homology with G-protein-coupled receptors (GPCRs) and build a hydrophobic cavity that is open towards the membrane and filled with phospholipids. The luminal domain extends between transmembrane helices 1 and 2, and consists of eight β-strands stabilized by two disulfide bonds.

Previous studies have called the N-terminal Wnt region the ‘thumb’ (from which the Wnt hairpin structures 1 and 2 extend, with hairpin 2 carrying the lipid modification) and the C-terminal region (carrying hairpin 3) the ‘index finger’, which together grab the cysteine-rich domain (CRD) of Frizzled receptors like a hand (Janda et al., 2012). To stay in this image, the Wnt ‘thumb’, when in complex with Evi/Wls, protrudes into the hydrophobic opening formed by the GPCR-like fold in such a way that the palmitoleate is inserted between Evi/Wls transmembrane helices 4 and 5 in the lipid bilayer. WNT8A and WNT3A adopt a very similar conformation in the reported models, although members of the WNT3 family have an extended N terminus compared with WNT8. Evi/Wls interacts at multiple positions with the bound WNT molecules, exemplified by a 2403 Å² buried surface between WNT8A and Evi/Wls (Nygaard et al., 2021). Analysis of surface conservation of human Wnts and Evi/Wls across species indicates that these interaction interfaces are highly similar between Wnts (Zhong et al., 2021). Whether Evi/Wls undergoes conformational changes during Wnt binding and how (and where) Wnt and Evi/Wls separate again remain to be analysed.

Intriguingly, the Wnt structure differs when in complex with Evi/Wls from the Frizzled CRD-associated structures reported earlier (Hirai et al., 2019; Janda et al., 2012). Whereas the Wnt hairpins 1 and 2 interact in the Wnt-CRD complexes, they appear separated by an Evi/Wls β-strand close to the ER membrane. Furthermore, all three hairpin loops are shifted by more than 15 Å (or 30-90°) between the positions reported in the Wnt- Frizzled CRD and Wnt-Evi/Wls complex, indicating high flexibility and possibly an induced fit mechanism, depending on the binding partner (Hirai et al., 2019; Janda et al., 2012; Nygaard et al., 2021; Zhong et al., 2021). Mutational and biochemical studies have determined that Wnt hairpin 3 is more crucial for interacting with the Frizzled CRD than for Wnt secretion (Nygaard et al., 2021). Notably, the Wnt lipid moiety localizes within the lipid bilayer when Wnts are in complex with Evi/Wls or Porcupine, but localizes outside the membrane when in complex with Frizzled; it remains unclear when and how this positional realignment is achieved (Liu et al., 2022; McGough et al., 2020; Nile et al., 2017; Nygaard et al., 2021; Zhong et al., 2021).

**Fig. 3.**
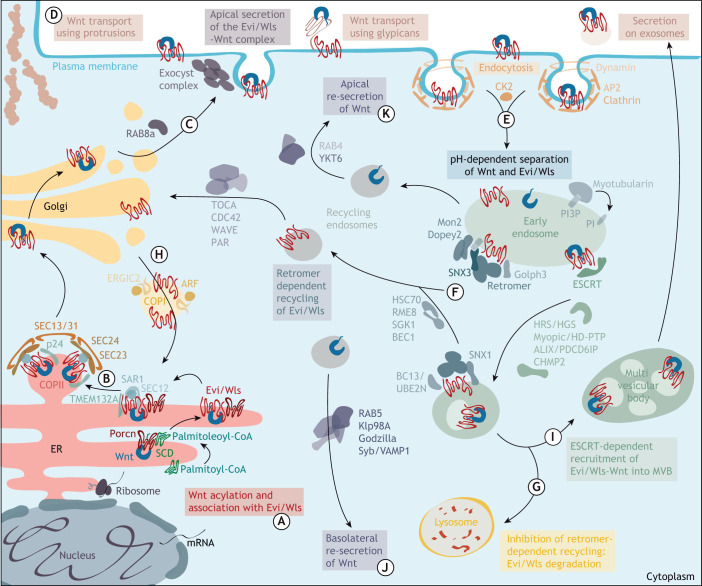
**Molecular mechanisms of Wnt export.** (A) Wnts are post-translationally modified with palmitoleate in the endoplasmic reticulum (ER) by the acyl-transferase Porcupine (Porcn) before engaging with their specific transport protein Evi/Wntless (Wls). (B) Wnt-Evi/Wls complexes are then packaged into COPII vesicles. (C) The Evi/Wls-Wnt complex then traverses the Golgi and reaches the plasma membrane, from where Wnts can be secreted. (D) Several different routes of intercellular Wnt transport have been described and potentially occur in a context-dependent manner. These routes include transport on cellular protrusions (cytonemes), glypicans or secretion on exosomes. (E) For efficient Wnt secretion, Evi/Wls needs to be endocytosed in a clathrin-dependent manner. (F) Via early endosomes, Evi/Wls can undergo retrograde transport back to the trans-Golgi network and the ER with the help of retromer, where it can associate with another Wnt molecule. (G) When retromer-dependent retrieval of Evi/Wls is inhibited, Evi/Wls is trafficked along the degradative endolysosomal route to lysosomes for degradation. (H) Evi/Wls is transported back to the ER via the RKEAQE C-terminal sequence. (I-K) Alternative routes of Wnt secretion include the packaging of the Evi/Wls-Wnt complex into intraluminal vesicles of multivesicular bodies (MVBs) and their secretion on exosomes (I) and basolateral (J) or apical (K) re-secretion of Wnts after trafficking through the endo-lysosomal system. Although depicted here in one summarising figure, the processes of Wnt export are likely context-, tissue and cell-type dependent. Refer to the main text for further molecular details.

After Wnt acylation and the binding of Wnt to Evi/Wls (Fig. 3A), the complex stays together and traverses the cellular protein secretion route at least until it reaches the cell membrane, from where several different export mechanisms have been described (Fig. 3) (reviewed by Routledge and Scholpp, 2019; Mittermeier and Virshup, 2022).

## Anterograde transport of the Evi/Wls-Wnt complex from the ER to the plasma membrane

The binding of Wnt to Evi/Wls in the ER membrane initiates recruitment into the early secretory pathway (Fig. 3B). Sun and colleagues used proteomic analysis to compare interaction partners of wild-type and a secretion-defective Evi/Wls variant in human HeLa cells and discovered that Evi/Wls interacts with SEC12 (also known as prolactin regulatory element-binding protein, PREB), a guanine nucleotide-exchange factor (GEF) and the small GTPase SAR1 at the ER membrane. SEC12 activates SAR1, which results in coat protein complex (COP) II vesicle assembly and Evi/Wls-Wnt complex export at ER exit sites (Sun et al., 2017). Evi/Wls-Wnt binding on the way from the ER to the plasma membrane is supported by the single-pass transmembrane protein TMEM132A, which interacts with itself and Evi/Wls (Li and Niswander, 2020).

Another set of essential players for Wnt secretion in the early secretory pathway are p24 family proteins, which are components of COPI and COPII-coated vesicles. Several members of this conserved transmembrane protein family (Éclair, Baiser, CHOp24, opossum and p24-1) are important for Wg or WntD secretion in *Drosophila* screens (Buechling et al., 2011; Li et al., 2015; Port et al., 2011). These insights have been corroborated by studies showing that the human p24 proteins TMED5 (an orthologue of opossum) and TMED3 also regulate Wnt signalling (Buechling et al., 2011; Duquet et al., 2014; Mishra et al., 2019). Although the exact function of p24 proteins for Wnt secretion is not fully understood, it has been proposed that they act as specific cargo receptors for COPII vesicles and convey contact between the ER luminal Wnts and cytosolic COPII proteins (Kinoshita et al., 2013; Pastor-Cantizano et al., 2016).

Members of the p24 family also aid the transport of glycosylphosphatidylinositol (GPI)-anchored proteins, a modification associated with Wnt ligand retention in the ER (Zoltewicz et al., 2009). However, it remains to be conclusively determined whether Wnt ligands themselves are modified with GPI-like modifications or whether it is rather the competition for p24 proteins that mediates this effect (Kinoshita et al., 2013; Zoltewicz et al., 2009).

The anterograde transport of Evi/Wls-Wnt from the Golgi to the plasma membrane is supported by Rab8A, but not Rab8B (Fig. 3C), as studied in the intestinal stem cell niche (Das et al., 2015). This dependency varies between cell type and Wnt ligands. In the absence of Rab8A, Evi/Wls was diverted to the endolysosomal compartment, presumably for lysosomal degradation (Das et al., 2015). Moti and colleagues used the retention using selective hook (RUSH) system to follow fluorescently labelled Wnts from the ER to the plasma membrane in human cells and noted that Wnt-loaded vesicles stopped for a moment close to the plasma membrane before being released; however, the underlying mechanisms remain to be elucidated (Moti et al., 2019).

## Wnt release from its producing cell

Several models exist to explain how Wnts are prepared for secretion and intercellular transport when approaching the plasma membrane (Fig. 3D,I). In general, two distinct possibilities have been demonstrated experimentally and are probably context dependent: Wnts can separate from Evi/Wls when reaching the plasma membrane, or they continue to travel together on vesicles (Nygaard et al., 2021). The latter could eventually result in an Evi/Wls-Wnt complex in the plasma membrane of the Wnt-receiving cell and would allow direct handover of Wnt from Evi/Wls to Wnt receptors, such as Frizzleds. Evidence for this model comes from analysis of transsynaptic communication in *Drosophila*, where Evi/Wls and Wnt were secreted on exosome-like vesicles at the neuromuscular junction (Korkut et al., 2009), a process that depended on Rab11, Syntaxin 1A and Myosin5 (Koles et al., 2012). The secretion of exosomes carrying active Wnts and Evi/Wls has also been demonstrated in *Drosophila*, mouse and human cells (Gross et al., 2012; Torres et al., 2021).

The alternative model, the separation of Wnts from Evi/Wls at the plasma membrane, is consistent with Wnt transport occurring with the help of lipid-binding carrier proteins and glypicans (Routledge and Scholpp, 2019). This release is associated with changes in pH along the secretory route; a low pH in vesicles is crucial for the successful release of WNT3A in human cell culture experiments (Coombs et al., 2010). However, this release could also occur after re-endocytosis from the plasma membrane (Linnemannstöns et al., 2020). Free diffusion of Wnts carrying a hydrophobic lipid modification in the extracellular space is highly unlikely, and several possibilities for intercellular Wnt ligand transport over short and long distances have been proposed. The recent reviews by Mittermeier and Virshup (2022), and Routledge and Scholpp (2019) provide an excellent overview of the many possibilities and the data supporting their occurrence. Regardless of the transport mechanism, it remains undetermined how handover to the receptors on the Wnt-receiving cell is achieved, a process that probably relies on several conformational changes.

## Recycling and retrograde transport of the Wnt cargo protein Evi/Wls

Wnt-less Evi/Wls at the plasma membrane is recycled back to the ER to allow association with further Wnt molecules and thus sustain efficient Wnt production (Gasnereau et al., 2011; Yu et al., 2014) (Fig. 3E). Recycling and retrograde transport of Evi/Wls depend on a conserved endocytosis motif within its third cytosolic loop (YEGL or YXXΦ, where Φ is a bulky amino acid). This motif is recognized by Clathrin adaptor protein 2 (AP-2) to initialize internalization through Clathrin and Dynamin (Gasnereau et al., 2011; Pan et al., 2008; Yamamoto et al., 2013), possibly with the help of casein kinase 2 (CK2) (de Groot et al., 2014). Mutating this YEGL motif prevents Evi/Wls internalization and results in its accumulation at the surface of HeLa cells (Gasnereau et al., 2011). The emerging Evi/Wls carriers are destined to fuse with early endosomes, where the cargo is sorted to the plasma membrane, undergoes lysosomal degradation or is recycled to the Golgi.

At the early endosome membrane, Evi/Wls is recruited into recycling vesicles by association with the trimer VPS26-VPS29-VPS35, a core component of the conserved protein coat complex retromer (Belenkaya et al., 2008; Franch-Marro et al., 2008; Kim et al., 2009; Pan et al., 2008; Port et al., 2008; Yang et al., 2008). It was initially suggested that this interaction could depend on a FLM motif in the third intracellular loop of Evi/Wls (Seaman, 2007; Yang et al., 2008), but this has not been experimentally tested. However, it was later shown that the interaction of vertebrate Evi/Wls with retromer depends on two Φ-X-[L/M] motifs at its C terminus (Varandas et al., 2016). Retromer is an essential mediator of protein retrieval from the endolysosomal pathway to the trans-Golgi network or the plasma membrane, and is essential in Wnt producing cells, as shown through studies in *C. elegans* around the same time that Evi/Wls was discovered (Coudreuse et al., 2006; Prasad and Clark, 2006) (Fig. 3F). More recently, functional links between efficient Wnt secretion and Evi/Wls recycling by retromer have been established, such as the propagation of mitochondrial stress between cells (Zhang et al., 2018b). The retromer subdomain VPS35 interacts with Golgi phosphoprotein 3 (GOLPH3) to support Evi/Wls recycling and Wnt signalling, thus promoting brain cancer (Lu et al., 2018). When retromer-dependent retrieval of Evi/Wls is inhibited, Evi/Wls is trafficked along the degradative endolysosomal route to lysosomes for degradation (Brown et al., 2020; de Groot et al., 2014; Gross et al., 2012; McGough et al., 2018; Shi et al., 2009; Zhang et al., 2018a; Zhu et al., 2015) (Fig. 3G).

Studies in *C. elegans* associate sorting nexin 1 (SNX1), as well as RME-8, HSC70, BEC-1 and the K63-linkage specific E3 ubiquitin ligase UBC-13, with the sorting of MIG-14/Evi/Wls (Ruck et al., 2011; Shi et al., 2009) (Fig. 3F). Depletion of UBC-13 leads to disruption of the endosomal subdomain organization and colocalization of MIG-14/Evi/Wls with an endosomal sorting complex required for transport (ESCRT) component (Zhang et al., 2018a). Furthermore, studies, initially in *Drosophila*, have established that retromer-dependent endosome-to-Golgi transport of Evi/Wls requires sorting nexin 3 (SNX3) binding to VPS35 at the early endosome (Harterink et al., 2011; Leneva et al., 2021; McGough et al., 2018; Port et al., 2011; Zhang et al., 2011). SNX proteins bind phosphatidylinositol-3-phosphate (PI3P) in lipid bilayers to anchor molecular complexes and shape membrane curvature in order to sequester cargo proteins into specific carriers (Harterink et al., 2011). The availability of PI3P can regulate SNX3-retromer-dependent endosomal trafficking; dephosphorylation of PI3P by myotubularins impairs Evi/Wls recycling and Wnt secretion in *C. elegans* and *Drosophila* (Silhankova et al., 2010). SNX3 knockout in mice, and respective defects in Wnt secretion due to faulty Evi/Wls recycling, result in fully penetrant cranial neural tube defects (Brown et al., 2020). This effect can be rescued by inducing Wnt signalling pharmacologically. Furthermore, a missense point mutation in SNX3 has been identified in a human individual with neural tube defects. Analogous cell culture experiments have since demonstrated that the mutated variant colocalized less with overexpressed Evi/Wls, indicating impaired capacity to retrieve Evi/Wls and to sustain Wnt export (Brown et al., 2020).

SNX3 lacks the curved Bin-Amphiphysin-Rvs (BAR) domain used by other members of the SNX family for membrane deformation and the formation of retromer-dependent cargo carriers (Leneva et al., 2021). Instead, structural analyses have revealed that SNX3 and VPS26-VPS29-VPS35 form ‘arch-like’ structures that directly bind membranes and induce bending through curved subcomplexes and oligomerization (Leneva et al., 2021). Interestingly, SNX-BAR proteins rescue Wnt secretion when Evi/Wls retrieval by SNX3-retromer and late endosomal maturation is inhibited. This implies that the preferential retrieval of Evi/Wls by SNX3-retromer is regulated through spatio-temporal separation from SNX-BAR proteins (Lorenowicz et al., 2014).

Based on live cell imaging studies and proteomic analysis in human cells, it has been suggested that Evi/Wls is recognized and enriched by SNX3 at early endosomes. It then uses RAB4-positive recycling endosomes (Fig. 3F) to travel back to the Golgi with the help of a conserved membrane remodelling complex composed of MON2 and DOPEY2, although the underlying molecular details remain to be elucidated (McGough et al., 2018; Molière et al., 2022; Zhao et al., 2020). In *C. elegans*, the transport of MIG-14/Evi/Wls from recycling endosomes to the trans-Golgi network requires the additional activity of TOCA, CDC-42, PAR and WAVE proteins (Bai and Grant, 2015). An earlier study linked Mon2 to serum- and glucocorticoid-inducible kinase 1 (SGK-1), the knockdown of which led to mislocalization of MIG-14/Evi/Wls, probably through an imbalance in sphingolipid levels and defects in Golgi-associated trafficking (Zhu et al., 2015).

After the relocation of Evi/Wls to the trans-Golgi network, the RKEAQE sequence at its C terminus mediates its transport back to the ER by endoplasmic reticulum-Golgi intermediate compartment protein 2 (ERGIC2) and the COPI vesicle regulator ADP-ribosylation factor (ARF) (Yu et al., 2014; Zhang et al., 2016) (Fig. 3H). Disruption of this sequence in human cells (e.g. by C-terminal tagging of Evi/Wls) prevents efficient Evi/Wls recycling and leads to its accumulation in the Golgi (Yu et al., 2014).

## Wnt secretion from polarized tissue

Wnt internalization and endocytosis have long been associated with regulating Wnt signalling, mostly in the Wnt-receiving cell (Dubois et al., 2001; Marois et al., 2006; Seto and Bellen, 2006). Several studies have shown that endocytosis of the Evi/Wls-Wnt complex at the plasma membrane is also an important step towards release of Wnt ligands on vesicles and for basolateral secretion from polarised cells (recently reviewed by Gross, 2021 and Mehta et al., 2021). The underlying molecular mechanisms have been primarily studied extensively in the wing imaginal disc, the precursor of the *Drosophila* wing, which consists of polarised epithelial cells. Wingless (Wg), the *Drosophila* WNT1 orthologue, is first presented on the apical cell membrane in an exocyst-dependent manner (Fig. 3C) before being endocytosed, transported to early endosomes and sorted into intraluminal vesicles in multivesicular bodies/late endosomes (Chaudhary and Boutros, 2019; Yamazaki et al., 2016). This sequestration depends on the ESCRT-associated proteins Myopic/Mop and HRS/HGS in *Drosophila* and mammalian cells, and reduction of Mop results in retention of Wg and Evi/Wls in early endosomes, leading to less basolateral secretion (Gross et al., 2012; Pradhan-Sundd and Verheyen, 2014). The connection between ESCRT and Wnt signalling is further strengthened by the observation that knockdown of the ESCRT-associated proteins Alix/PDCD6IP and CHMP2B reduces Wnt signalling activity in HEK293T cells (Linnemannstöns et al., 2020). Other mediators of Wnt transcytosis in the wing imaginal disc are Godzilla, an E3 ubiquitin ligase that acts together with the SNARE protein Synaptobrevin (Syb, human VAMP1) (Linnemannstöns et al., 2020; Yamazaki et al., 2016), and the kinesin motor Klp98A, the absence of which in basolateral Wnt secretion does not affect extracellular Wnt levels (Witte et al., 2020) (Fig. 3J).

The endocytosed pool of Wnts could serve as a ligand reservoir that can be re-released if required by a mechanism involving the v-SNARE Ykt6 and Rab4 recycling endosomes, but not Evi/Wls (Linnemannstöns et al., 2020; Witte et al., 2020) (Fig. 3K). The separation of Wg from Evi/Wls after apical endocytosis has been proposed to happen in acidified endosomes, after which Evi/Wls is recycled to the trans-Golgi network and Wg travels back to the plasma membrane (Coombs et al., 2010; Linnemannstöns et al., 2020).

Furthermore, it has been suggested that internalization of Wg is important for the interaction of Wg with its receptor DFz2 (Frizzled) in endosomes, which could potentially also evoke cell-autonomous signalling (Hemalatha et al., 2016; Marois et al., 2006; Seto and Bellen, 2006). How Wg is recognized for internalization remains to be explored. Several studies have suggested that glypicans, such as Dally-like, could facilitate internalization and transcytosis in a GPI anchor-dependent manner (Gallet et al., 2008). The internalization of Wg is independent of Dynamin in the fly (Hemalatha et al., 2016), whereas Dally-like or Evi/Wls internalization depends on Dynamin in the fly or mammals, respectively (Gallet et al., 2008; Gasnereau et al., 2011), indicating that there are distinct mechanisms that possibly differ between Wnt-producing and -receiving cell.

These insights generated in the *Drosophila* wing were corroborated by studies in mammalian polarised epithelial cells, in which Wnt1 and Wnt3a were secreted apically and basolaterally, and depended on Evi/Wls recycling, whereas Wnt11 was secreted only apically, independent of Evi/Wls recycling (Yamamoto et al., 2013, 2017).

## Evi/Wls is degraded in the absence of Wnt ligands

**Fig. 4.**
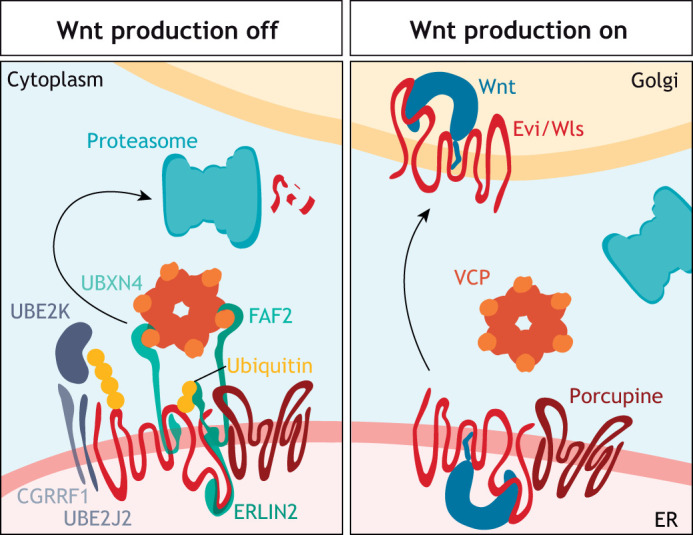
**Evi/Wls is degraded by the proteasome in the absence of Wnt ligands.** Evi/Wls is destined for proteasomal degradation in Wnt-producing cells when no Wnts are being secreted. In the endoplasmic reticulum (ER) membrane, Evi/Wls binds ER lipid raft-associated protein 2 (ERLIN2) before being ubiquitinylated by the E2 ubiquitin-conjugating enzymes UBE2J2, UBE2K and UBE2N (not shown), and the E3 ubiquitin ligase cell growth regulator with RING finger domain protein 1 (CGRRF1). Ubiquitylated Evi/Wls is removed from the ER membrane and delivered to the proteasome with the help of the AAA ATPase valosin-containing protein (VCP; also known as p97 or Cdc48), which is recruited to the ER membrane through Fas-associated factor 2 (FAF2; also known as UBXD8) and UBXN4. Once Wnt production is activated, lipid-modified Wnts are transferred from the acyl transferase Porcupine to their cargo protein Evi/Wls and the Evi/Wls-Wnt complex travels to the Golgi and the plasma membrane.

To our current knowledge, Evi/Wls is the exclusive carrier of most Wnt ligands. Nevertheless, it has been unclear how Evi/Wls protein levels are adjusted to accommodate differentially expressed Wnt ligands, a total of 19 in the human genome, with many different expression patterns. This question was addressed, in part, after Glaeser and colleagues observed that, in colon adenocarcinoma samples, WNT3 expression positively correlates with Evi/Wls protein abundance but not with Evi/Wls mRNA expression, suggesting post-translational mechanisms adapt Evi/Wls to Wnt protein levels (Glaeser et al., 2018). In-depth biochemical studies, candidate screening and proteomic workflows were used to subsequently discover that Evi/Wls is an endogenous substrate of ER-associated degradation (ERAD), a molecular pathway that mediates the ubiquitylation and degradation of ER-resident proteins (Fenech et al., 2020; Glaeser et al., 2018; Wolf et al., 2021) (Fig. 4). Evi/Wls is constantly produced in Wnt-secreting cells and immediately degraded in the absence of lipid-modified Wnts or after chemical inhibition of Porcupine (Glaeser et al., 2018; Wolf et al., 2021). The degradation of Evi/Wls depends on its ubiquitylation by the ER membrane-associated E3 ubiquitin ligase CGRRF1 and the E2 ubiquitin conjugating enzymes UBE2J2, UBE2K and UBE2N, at multiple amino acid positions (Fenech et al., 2020; Glaeser et al., 2018; Wolf et al., 2021). Genetically deleting the ER membrane-associated protein ERLIN2 markedly reduces Evi/Wls ubiquitylation, indicating that this protein is important to select Evi/Wls for ubiquitylation and degradation (Wolf et al., 2021). The physical removal of Evi/Wls from the ER membrane is performed by the cytoplasmic AAA ATPase VCP/p97, an essential player in ERAD, which is docked to the ER membrane with the help of FAF2 and UBXN4 (Glaeser et al., 2018; Wolf et al., 2021). Several of the described components of the Evi/Wls ‘destruction complex’ regulate Wnt secretion in biochemical assays and are associated with cancer progression, emphasizing the importance of studying the underlying mechanisms in more depth (Li et al., 2018; Wang et al., 2012a,b; Wolf et al., 2021).

The post-translational regulation of Evi/Wls protein abundance by Wnt expression can potentially be used to help explain earlier findings: it was, for example, observed that depletion of β-catenin from nail epithelium led to the downregulation of Wnts and Evi/Wls protein in this tissue (Takeo et al., 2013, 2016). As Wnt ligands are downstream targets of β-catenin transcriptional regulation in keratinocytes (Deschene et al., 2014), the loss of Evi/Wls could be a secondary effect. Another study in the *Drosophila* wing imaginal disc showed that depletion of Porcupine led to increased Wg levels at the dorsal/ventral boundary but to reduced Evi/Wls levels, an observation that is in line with the requirement of lipid-modified Wnts to stabilize Evi/Wls (Zhang et al., 2016).

Many other components of the Wnt signalling pathways are regulated by post-translational modification with ubiquitin and proteasomal degradation. The most prominent example is, of course, β-catenin itself (reviewed by Söderholm and Cantù, 2021), but also Frizzled receptors and, more recently, the non-canonical Vangl proteins have been found to be regulated by the ubiquitin-proteasome system (Feng et al., 2021; Rim et al., 2022). Although the degradation of misfolded proteins is essential for avoiding cellular stress by maintaining a healthy proteome and saving resources, removing functional proteins has an important regulatory function (Hegde and Ploegh, 2010). As such, it is believed that the regulation of Evi/Wls protein abundance through ERAD contributes to the dynamic adaptation of the Wnt-producing cell to varying levels of Wnt expression in two ways. On the one hand, it allows cells to secrete Wnts directly after their production, without the need to translate the Wnt transport protein Evi/Wls first, and thus allows a more immediate response to Wnt-stimulating cues and reduces the accumulation of lipid-modified Wnt protein in the ER. On the other hand, it raises the possibility that the post-translational regulation of Evi/Wls adds another layer of regulation to Wnt transport and might help the convergence of several cellular signals to regulate Wnt signalling (Glaeser et al., 2018; Wolf et al., 2021).

Despite the mechanisms that have been discovered, essential questions about the described processes remain unanswered: for example, it remains unknown whether Evi/Wls is cleaved before its removal or whether a channel protein is needed to assist detachment from the ER membrane. Furthermore, the exact ubiquitylation sites of Evi/Wls and their functional relevance remains to be investigated, especially in view of protein trafficking, which can be regulated by UBE2N (Zhang et al., 2018a).

## Wnt export, organ development and disease

In recent years, several studies have explored the contribution of Wnt export to stem cell physiology, embryogenesis, and the development of bone and teeth (reviewed by Du et al., 2021), lung, liver, reproductive organs, synapses and the central nervous system, among others (Table 1). A very well-studied system is the mammalian intestine; importantly, while epithelial cells in the intestine (most notably Paneth cells in the small intestine) do express Wnts, several pieces of evidence suggest that the Wnts expressed by underlying stromal cells are enough to sustain intestinal stem cells and a healthy gut (de Groot et al., 2014; Farin et al., 2012; Gao et al., 2019; Kabiri et al., 2014; Valenta et al., 2016). Nevertheless, the purpose of multiple Wnt sources in the intestine has remained elusive. Recent evidence indicates that epithelial Wnts are essential for healing the mouse gut after colitis-like injury, showing that multiple Wnt sources contribute to maintain high Wnt levels (Das et al., 2022).

**Table 1.**
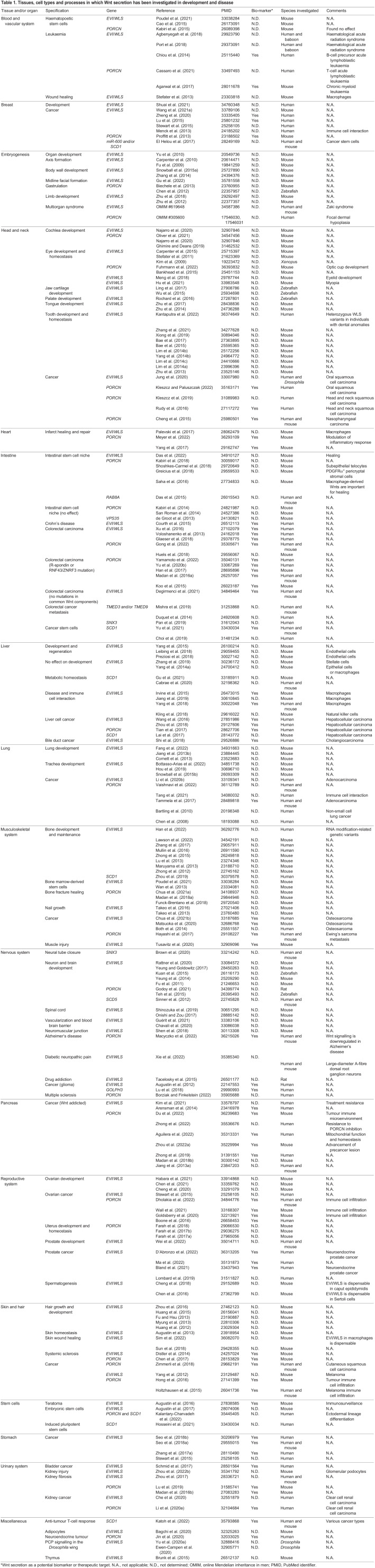
Tissues, cell types and processes in which Wnt secretion has been investigated in development and disease

Given the importance of Wnt signalling during embryonic development and for stem cell maintenance (reviewed by Steinhart and Angers, 2018), it is not surprising that Porcupine and Evi/Wls are also associated with congenital disorders. Mutations in the *PORCN* gene are associated with focal dermal hypoplasia (also called Goltz-Gorlin syndrome, OMIM #305600), a rare X-linked syndrome affecting the skin, skeleton, teeth and eyes of females and, in very rare cases, males (Frisk et al., 2018; Grzeschik et al., 2007; Wang et al., 2007). The *WLS* genetic locus has been connected to bone mineral density and osteoporosis (Mullin et al., 2016; Zhang et al., 2017b). Nevertheless, the number of reported cases of congenital mutations in the genes encoding Porcupine or Evi/Wls is rather low, probably reflecting how essential the underlying processes are for the development of human life and the variability of the resulting phenotype, which makes it more difficult to find a unifying characteristic or genetic cause. Therefore, it requires the careful analysis of large datasets to find causative mutations to explain structural birth defects associated with Wnt secretion, as was recently performed by Chai et al. (2021): after reviewing over 20,000 families from worldwide databases and extensive genetic analysis to find pathogenic variants, five families with mutations in the *WLS* gene were discovered. Biochemical and cell biological analysis proved that the mutations reduced Evi/Wls protein stability and Wnt-secretion capacity. The affected children presented with defects of the face, brain, skeleton, kidney and heart. Using mouse models reflecting the human mutations, the authors could ameliorate these birth defects by treating the pregnant mice with a GSK3 inhibitor, thus activating the Wnt/β-catenin transcriptional response. Whereas this approach shows the potential preventability of some disorders, the authors are right to caution that such treatments require a thorough examination of potential risks to mother and child (Chai et al., 2021).

Evi/Wls was also implicated in very complex disease traits, such as gastrointestinal dysfunction in autism (Fröhlich et al., 2019), diabetic neuropathic pain (Xie et al., 2022) and drug addiction. For the latter, several studies have explored the underlying molecular mechanisms and could show Evi/Wls protein interaction with opioid receptors and other reward and addiction-related proteins (Petko et al., 2018). Mechanistically, Evi/Wls interacts with opioid receptors at the cell surface, presumably inhibiting its recycling and preventing Wnt secretion (Jin et al., 2010a). The role of Evi/Wls in drug addiction has also been corroborated by behavioural studies analysing the motivation for heroin in rats (Tacelosky et al., 2015).

More frequently, aberrant Wnt secretion is associated with cancer progression or tumour immune response in various tissues (Table 1) (reviewed by Galluzzi et al., 2019, and Katoh and Katoh, 2022). Mutations in Wnt pathway components are especially abundant in colorectal cancer, affecting nearly all clinical cases (Cancer Genome Atlas Network, 2012; Yaeger et al., 2018; Zehir et al., 2017; Zhan et al., 2017). However, as most of these mutations induce Wnt signalling downstream of Wnt secretion, the regulation of Wnt export is not usually considered to be a relevant treatment option, although colon cancer cells secrete and respond to Wnt ligands (Voloshanenko et al., 2013). A recent publication described a subset of human colon cancer cases without mutations in core Wnt components and a respective mouse model that required autosecreted Wnts for survival (Degirmenci et al., 2021). Furthermore, another subset of colon cancers with mutations in RNF43/ZNRF3 or R-spondins 2/3 require secreted Wnts for proliferation (Flanagan et al., 2022). These tumours would potentially respond to inhibition of Wnt export and emphasise the importance of molecular classification of tumours to find suitable treatment options (Flanagan et al., 2022).

## Monitoring and manipulating Wnt export: Evi/Wls as a biomarker

Whereas many of the factors associated with Wnt export have a general function in protein transport between organelles, Evi/Wls and Porcupine are apparently Wnt specific. Therefore, targeting these proteins is currently the most feasible way to regulate Wnt secretion and downstream signalling events across a broad spectrum of Wnt ligands. As there are no commercial small molecules binding Evi/Wls, this protein is mostly targeted using genetic tools and is often exploited in animal models to analyse tissue or cell type-specific effects of Wnt secretion. A summary of Evi/Wls and Porcupine animal models in the mouse, *C. elegans* and *Drosophila* can be found in Tables S1, S2 and S3, respectively.

The recent elucidation of the Evi/Wls structure revealed homology to G-protein-coupled receptors with a functional pocket and raised the possibility of developing specific inhibitors in the future (Nygaard et al., 2021). Such specific small molecule inhibitors have already been successfully developed to target the enzymatic function of Porcupine and are amongst the most potent agents to target Wnt signalling. These agents are invaluable tools in research and are tested in clinical trials to treat various cancers. Nevertheless, systemic application of Porcupine inhibitors has been associated with severe unwanted on-target effects as a consequence of the indispensability of Wnt secretion for stem cell and tissue homeostasis, e.g. resulting in bone toxicity (reviewed by Jung and Park, 2020; Neiheisel et al., 2022; Parsons et al., 2021; Zhang and Lum, 2018).

Evi/Wls protein abundance is relative to the available Wnt proteins in the Wnt-secreting cell through a direct, post-translational mechanism (Glaeser et al., 2018; Wolf et al., 2021). This functional engagement allows targeted monitoring and manipulation of Wnt signalling activity. Therefore, Evi/Wls has been suggested to be used as a biomarker for Wnt expression and for disease progression in cancers affecting breast, liver, pancreas, gastrointestinal tract, brain, bone and prostate (Table 1). Using Evi/Wls as a biomarker could help to predict therapy resistance, to identify individuals for Wnt-targeting therapy or to identify the tissue of origin (Augustin et al., 2012; Bland et al., 2021; Chua et al., 2021b; Kim et al., 2021; Ma et al., 2022; Seo et al., 2018a,b; Shi et al., 2018; Shuai et al., 2021; Zheng et al., 2020; Zhou et al., 2018). For example, Ma and colleagues recently treated a mouse model created using a Evi/Wls-positive pancreatic cancer patient-derived xenograft with Porcupine inhibitors and thus reduced tumour growth (Ma et al., 2022). The potential use of Evi/Wls as a biomarker extends beyond its application in cancer research after a recent study associated the expression signature of several genes, including *WLS*, with chronic neuropathic pain (Islam et al., 2021). The Evi/Wls specific monoclonal antibody YJ5 has been suggested for use as an immunohistochemical marker of the osteogenic lineage (Chua et al., 2021b).

## Conclusions and future perspectives

Delineating signalling cascades, their interaction and regulation allows us to better understand cellular activity and to draw informed conclusions about their deregulation in diseases. Many developmental signalling programmes, including Wnt signalling, are reactivated during malignant transformation and offer potential targets for tumour therapy. Therefore, studying the mechanisms underlying Wnt ligand export and supporting these insights with structural data can lead to the development of novel molecular tools for the Wnt field of study and for further clinical advancements. The acyltransferase Porcupine and the Wnt transport protein Evi/Wls are promising candidate cellular reporters for detecting active Wnt secretion or novel biomarkers for classifying diseases. Hence, it will be crucial to better define all Wnt-dependent and -independent effects of Evi/Wls and Porcupine to understand possible unwanted effects originating from their manipulation.

Furthermore, our comprehension of Evi/Wls and Porcupine-independent Wnt secretion should be deepened. Most famously, the *Drosophila* WntD is neither lipid modified nor secreted with the help of Evi/Wls (Ching et al., 2008). There is also evidence for Evi/Wls-independent secretion of Wnts in vertebrates (Wu et al., 2015; Yamamoto et al., 2013) but the underlying molecular mechanisms remain to be explored. Whereas the lipid-modification of Wnts seems to be crucial for their interaction with the transport protein Evi/Wls (Glaeser et al., 2018), the existence of non-acylated Wnt agonists, such as Norrin (Chang et al., 2015), Wnt Surrogate (Janda et al., 2017) or Frizzled and LRP5/6 agonist (FLAg) (Tao et al., 2019), argues against the strict requirement of this lipid moiety for the activation of downstream Wnt signalling. Accordingly, there are reports in animal and cell culture models describing Porcupine and lipid-independent Wnt functions (Rao et al., 2019; Speer et al., 2019).

Further areas of interest for future research are the involvement of ubiquitylation in Wnt transport (Glaeser et al., 2018; Wolf et al., 2021; Yamazaki et al., 2016; Zhang et al., 2018a) and the various intersections between Wnt signalling and lipid metabolism (Bagchi et al., 2020; Katanaev et al., 2008; Nygaard et al., 2021; Olzmann and Carvalho, 2019). Importantly, the relationship between secreted Wnts and the resulting signalling strength in target cells should be addressed carefully because a recent study in *Drosophila* has found that the induced signalling is, in large part, independent of how much of the signalling molecule is initially produced (Hatori et al., 2021). Despite the many advances in our understanding of Wnt secretion and signal transduction, important questions remain to be answered, especially regarding the exquisite spatio-temporal regulation of Wnt ligand expression and Wnt ligand transfer between transport proteins and receptors.

## Supplementary Material

10.1242/develop.201352_sup1 Supplementary information
