# Supplementary material for: The role of Evi/Wntless in exporting Wnt proteins
Source: Development. 2023 Feb 13;150(3):dev201352. doi: 10.1242/dev.201352 (PMC10112924; doi:10.1242/dev.201352)
Supplement: Supplementary information [file develop-150-201352-s1.pdf]

Full references are provided in the Review article

Table S1. Mouse models for investigating Wnt secretion

Target	Name	Features	Reference	PMID	Comments
Evi/Wntless/GPR177 (Wls)	$Wls^{fl/fl}$	*loxP* sites inserted in the 5′ untranslated region before the ATG and upstream of exon 2 of the *Wls* gene	Carpenter et al. (2010)	20614471	129S-$Wls^{tm1.1Lan}$/J; Jackson Laboratory Strain 012888; RRID:IMSR_JAX:012888
	$Gpr177^{fl/fl}$	*loxP* sites flanking exon 3 of the *Wls* gene	Fu et al. (2011)	21246653	STOCK $Wls^{tm1.1Whsu}$/J; Jackson Laboratory Strain 027484; RRID:IMSR_JAX:027484
	$Wls^{c/c}$	*loxP* sites flanking exon 3 of the *Wls* gene	Zhu et al. (2012)	22377357	N.A.
	$Evi^{fl/fl}$	*loxP* sites flanking exon 3 of the *Wls* gene	Augustin et al. (2013)	23918954	N.A.
	ROSA26::Evi-YFP	β-geo reporter flanked by *loxP* sites followed by *Wls* with a C-terminal YFP inserted in the ROSA26 locus	Augustin et al. (2012)	22147553	Evi/Wls gain-of-function construct
	$Gpr177^{lacZ}$	β-geo reporter inserted between exon 9 and 10 of the *Wls* gene. The knock-in allele encodes a Wls-β-gal reporter protein. Heterozygous mice are healthy and viable; homozygous animals die during early embryogenesis.	Fu et al. (2009); Yeung et al. (2014)	19841259; 25209290	Based on the mutant ES cell line RRJ545 (Bay Genomics); B6.129P2-$Wls^{Gt(RRJ545)Byg}$/WhsuJ; Jackson Laboratory Strain 024775; RRID:IMSR_JAX:024775
	Wls p.Y392C and *Wls* p.Y478C	Knock-in mice generated using CRISPR/Cas9. Heterozygous mice are healthy and viable, homozygous animals die around the time of birth	Chai et al. (2021)	34587386	Animal models mimicking human Zaki syndrome caused by *WLS* mutations
Porcupine (*Porcn*), X-linked	$Porcn^{lox}$	*loxP* sites flanking exons 2 and 3 of the *Porcn* gene	Barrott et al. (2011)	21768372	N.A.
	Porcn-ex3-7flox	*loxP* sites flanking exons 3 to 7 of the *Porcn* gene	Liu et al. (2012)	22412863	129S-$Porcn^{tm1.1Vdv}$/J; Jackson Laboratory Strain 020994; RRID:IMSR_JAX:020994
	$Porcn^{fl}$	*loxP* sites flanking exon 3 of the *Porcn* gene	Biechele et al. (2013)	23760955	N.A.

PMID, PubMed identifier.

Table S2. *Caenorhabditis elegans* models for investigating Wnt secretion

Target	Name	Features	Reference	PMID	Comments
Evi/Wntless/MIG-14 (*mig-14*)	ga62	Allele with p.H329Y substitution; viable as homozygous strain and partial disruption of MIG-14 function (Pan et al., 2008)	Eisenmann and Kim (2000)	11063687	WormBase ID: WBVar00145405; strain EW12
	gm2	Allele, mutation not specified; maternal-effect lethality and severely reduced MIG-14 activity (Pan et al., 2008)	Pan et al. (2008)	18160346	WormBase ID: WBVar00146311
	k124	Allele, mutation not specified	Harris et al. (1996)	8898225	WormBase ID: WBVar00088195
	mu71	Allele, mutation not specified; viable as homozygous strain and partial disruption of MIG-14 function (Pan et al., 2008)	Harris et al. (1996)	8898225	WormBase ID: WBVar00089206; strain CF367
	or78	Allele with 165 bp deletion in-frame loss of amino acids 374-428; maternal-effect lethality and severely reduced MIG-14 activity (Pan et al., 2008)	Thorpe et al. (1997)	9288749	WormBase ID: WBVar00094674; strain EU404
	tm2919	Allele with 472 bp deletion	*C. elegans* Deletion Mutant Consortium (2012)	23173093	WormBase ID: WBVar00251731
	zu21	Allele, mutation not specified	Bei et al. (2002)	12110172	WormBase ID: WBVar00275481
	expr9989_Ex	Transgene, MIG-14-GFP fusion protein under the *egl-20* promoter egl-20 is a *C. elegans* Wnt	Pan et al. (2008)	18160346	[Pegl-20::mig-14::gfp]; WormBase ID: WBTransgene00031328
	gmEx508	Transgene, MIG-14-GFP fusion protein under the *egl-20* promoter egl-20 is a *C. elegans* Wnt	Pan et al. (2008)	18160346	[Pegl-20::mig-14::gfp; dpy-30:: NLS::DsRed]; WormBase ID: WBTransgene00007903
	huIs71	Transgene, MIG-14-GFP fusion protein under the *mig-14* promoter	Yang et al. (2008)	18160347	[Pmig-14::mig-14::gfp]; WormBase ID: WBTransgene00009586
	huIs72	Transgene, MIG-14-GFP fusion protein under the *mig-14* promoter	Yang et al. (2008)	18160347	[Pmig-14::MIG-14::GFP::unc-54 3'UTR]; WormBase ID: WBTransgene00005942
	huSi2	Transgene, MIG-14-GFP fusion protein under the *mig-14* promoter single copy transgene	Silhankova et al. (2010)	21076391	[Pmig-14::mig-14::gfp]; WormBase ID: WBTransgene00009616
	pwIs765	Transgene, MIG-14-GFP fusion protein under the *vha-6* promoter intestinal expression	Shi et al. (2009)	19763082	[Pvha-6::MIG-14::GFP]; WormBase ID: WBTransgene00009510
	pwIs792	Transgene, MIG-14-GFP fusion protein under the *pie-1* promoter germline expression	Shi et al. (2009)	19763082	[Ppie-1::MIG-14::GFP; unc-119+)]; WormBase ID: WBTransgene00009511
	pwIs911	Transgene, MIG-14-GFP fusion protein under the *vha-6* promoter intestinal expression	Gleason et al. (2016)	27630264	[Pvha-6::MIG-14::GFP]; WormBase ID: WBTransgene00023437
	pwIs995	Transgene, MIG-14-GFP fusion protein under the *snx-1* promoter retromer associated sorting nexin	Norris et al. (2017)	28053230	[psnx-1::mig-14::gfp-unc54 3'UTR-cb-unc-119]; WormBase ID: WBTransgene00023810
	twnEx303	Transgene, MIG-14-mCherry fusion protein under the *Ser-2.3* promoter PVD neuron expression	Liao et al. (2018) (several other constructs)	29673481	[Pser-2.3::mig-14::mCherry, Pgcy-8::mCherry]; WormBase ID: WBTransgene00025251
Porcupine/MOM-1 (mom-1)	or10	Allele, mutation not specified	Thorpe et al. (1997)	9288749	WormBase ID: WBVar00094648; strains EU308 and EU361
	or46	Allele, mutation not specified	Thorpe et al. (1997)	9288749	WormBase ID: WBVar00094667; strain EU307
	or65	Allele, mutation not specified	Thorpe et al. (1997)	9288749	WormBase ID: WBVar00094671
	or70	Allele, mutation not specified	Thorpe et al. (1997)	9288749	WormBase ID: WBVar00094672

Continued

Table S2. Continued

Target	Name	Features	Reference	PMID	Comments
	or83	Allele, mutation not specified	Thorpe et al. (1997)	9288749	WormBase ID: WBVar00094677
	ne117	Allele, mutation not specified	Rocheleau et al. (1997)	9288750	WormBase ID: WBVar00090960
	se2	Allele, mutation not specified	Rocheleau et al. (1997)	9288750	WormBase ID: WBVar00242723
	zu188	Allele, mutation not specified	Rocheleau et al. (1997)	9288750	WormBase ID: WBVar00275516
	zu204	Allele, mutation not specified	Rocheleau et al. (1997)	9288750	WormBase ID: WBVar00275524
	zu237	Allele, mutation not specified	Rocheleau et al. (1997)	9288750	WormBase ID: WBVar00275535

Based on information available at WormBase, Version: WS283.
PMID, PubMed identifier.

Table S3. *Drosophila melanogaster* models of Wnt secretion

Target	Name	Features	References	FlyBase ID	Comments
Evi/wntless (*wls*)	$wls^{EY01593}$	Allele, transposable element insertion using P element activity. P{EPgy2} element insertion 152 bp upstream of the *wls* gene start codon, resulting in wing defects.	Bartscherer et al. (2006) PMID: 16678096; Bellen et al. (2004) PMID: 15238527	FBal0148189	Stock number 15363 BDSC: y^1 w^{67c23}; P{EPgy2} $wls^{EY01593}$
	$wls^{EY11001}$	Allele, transposable element insertion using P element activity. P{EPgy2} element insertion upstream of the *wls* gene start codon.	Bellen et al. (2004) PMID: 15238527	FBal0160207	Stock number 20651 BDSC: y^1 w^{67c23}; P{EPgy2} $wls^{EY11001}$
	wls^{c06300}	Allele, transposable element insertion using piggyBac activity. PBac{PB} element insertion in the 3′ UTR.	Thibault et al. (2004) PMID: 14981521; Bellen et al. (2011) PMID: 21515576	FBal0162818	Stock number 17756 BDSC: w^{1118}; PBac {PB}wls^{c06300}/ TM6B, Tb^1
	wls^{c06303}	Allele, transposable element insertion using piggyBac activity	Thibault et al. (2004) PMID: 14981521; Bellen et al. (2011) PMID: 21515576	FBal0182819	N.A.
	wls^{c05902}	Allele, transposable element insertion using piggyBac activity	Thibault et al. (2004) PMID: 14981521; Bellen et al. (2011) PMID: 21515576	FBal0182820	N.A.
	wls^1	Allele, lethal deletion of 47 bp of the wls gene, leading to a frameshift at amino acid 29	Bänziger et al. (2006) PMID: 16678095	FBal0194734	N.A.
	wls^2	Allele, lethal point mutation resulting in amino acid change (p.P250S)	Bänziger et al. (2006) PMID: 16678095	FBal0194735	N.A.
	$wls^{\alpha Tub84B.PB}$	A αTub84B promoter drives expression of a wls transgene isoform A	Bänziger et al. (2006) PMID: 16678095	FBal0194736	N.A.
	$wls^{UAS.Tag:HA}$ (UAS-wls)	Transgenic construct containing *wls* isoform A with a C-terminal hemagglutinin (HA) tag	Bänziger et al. (2006) PMID: 16678095	FBal0194737	N.A.
	$wls^{\alpha Tub84B.Tag:HA}$	A αTub84B promoter drives expression of a wls transgene isoform A with a C-terminal hemagglutinin (HA) tag	Bänziger et al. (2006) PMID: 16678095	FBal0194738	N.A.
	$wls^{UAS.EGFP}$	Transgenic construct containing *wls* with a C-terminal green fluorescent protein (EGFP) tag	Bartscherer et al. (2006) PMID: 16678096	FBal0194740	N.A.
	evi^2	Loss-of-function allele, lethal deletion of 771 bp of the *wls* gene, affecting 209 N-terminal amino acids	Bartscherer et al. (2006) PMID: 16678096	FBal0194741	N.A.
	wls^{GD2418}	RNAi construct targeting *wls* GAL4 system	Dietzl et al. (2007) PMID: 17625558	FBal0210893	Stock numbers v5214:w^{1118}; P{GD2418} v5214 and v5215: w^{1118}; P{GD2418} v5215 VDRC
	wls^{7E4}	Loss-of-function allele, lethal point mutation resulting in premature stop codon p.W524X in isoform PA and p.W492X in isoform PB	Goodman et al. (2006) PMID: 17108000	FBal0211022	N.A.

Continued

Table S3. Continued

Target	Name	Features	References	FlyBase ID	Comments
	wls$^{01593\text{-}1}$	Transgenic construct containing *wls* with a C-terminal V5 tag	Franch-Marro et al. (2008) PMID: 18193037	FBal0221636 FBal0211024	N.A.
	wls$^{UAS.Tag:V5}$	Allele, lethal deletion that removes the 5′ UTR and part of exon 1 of the *wls* gene	Goodman et al. (2006) PMID: 17108000		
	wls^{KK101700}	RNAi construct targeting *wls* GAL4 system	Dietzl et al. (2007) PMID: 17625558	FBal0231429	Stock number v103812 VDRC: P{KK101700} VIE-260B
	wls$^{UAS.ORF.GW.Tag:HA}$	Transgenic construct containing the full-length *wls* open reading frame with a C-terminal hemagglutinin (HA) tag	Bischof et al. (2013) PMID: 23637332; Bischof et al. (2014) PMID: 24922270	FBrf0243696	Stock number F003525 FlyORF: M{UAS-wls.ORF.3xHA. GW}ZH-86Fb
	wls$^{hs.Tag:V5}$ (hs-wls-V5)	Transgenic construct containing *wls* with a C-terminal V5 tag	Belenkaya et al. (2008) PMID: 18160348	FBal0244076	N.A.
	wls$^{UAS.cBa}$	Transgenic construct containing full length *wls* cDNA	Belenkaya et al. (2008) PMID: 18160348	FBal0244077	N.A.
	wls$^{UAS.Tag:V5.cBa}$	Transgenic construct containing *wls* with a C-terminal V5 tag	Belenkaya et al. (2008) PMID: 18160348	FBal0244078	N.A.
	wls$^{UAS.cBa.Tag:HA}$ (UAS-wls-HA)	Transgenic construct containing *wls* with a C-terminal hemagglutinin (HA) tag	Belenkaya et al. (2008) PMID: 18160348	FBal0244079	N.A.
	wls$^{\Delta 1.lacZ}$	Transgenic construct containing *wls* in which the first exon has been replaced by EcoI\lacZ	Herr and Basler (2012) PMID: 22108505	FBal0267625	N.A.
	wls^{+tGa}	A wild type genomic rescue fragment	Gasnereau et al. (2011) PMID: 22027831	FBal0268422	N.A.
	wls$^{t.AEGL}$	Transgenic *wls* construct containing an amino acid substitution p.Y435A, leading to impaired recycling of wls from the plasma membrane and wing defects	Gasnereau et al. (2011) PMID: 22027831	FBal0268423	N.A.
	wls$^{UAS.cKa.Tag:HA}$	Transgenic construct containing *wls* isoform A with a C-terminal hemagglutinin (HA) tag	Koles et al. (2012) 22437826	FBal0269140	N.A.
	wls$^{g80.Tag:OLLAS}$	Transgenic construct containing *wls* with an OLLAS tag after amino acid 474	Beckett et al. (2013) PMID: 23035643	FBal0293732	N.A.
	wls$^{dsRNA.UAS.cVa}$	RNAi construct targeting *wls* GAL4 system	Corrigan et al. (2014) PMID: 25154396	FBal0301273	N.A.
	wls$^{gRNA.snRNA:U6:96Ac}$	An ubiquitous promoter drives expression of one Cas9-specific gRNAs targeting *wls*	Port et al. (2014) PMID: 25002478	FBal0302200	Cas9 mediated mutagenesis. Targeting the signalling sequence.
	wls^{G32C}	Allele, nucleotide replacement using CRISPR/ Cas9 g.G32C resulting in p.G11A	Port et al. (2014) PMID: 25002478	FBal0302201	Cas9 mediated mutagenesis. Used to destroy PAM at this position.
	wls$^{gRNA.U6:3.FP410}$	An ubiquitous promoter drives expression of one Cas9-specific gRNAs targeting *wls*	Port et al. (2015) PMID: 25999583	FBal0364035	Cas9 mediated mutagenesis
	wls$^{gRNA.U6:3.FP412}$	An ubiquitous promoter drives expression of one Cas9-specific gRNAs targeting *wls*	Port et al. (2015) PMID: 25999583	FBal0364036	Cas9 mediated mutagenesis
	wls$^{gRNA.U6:3.FP512}$	An ubiquitous promoter drives expression of one Cas9-specific gRNAs targeting *wls*	Port et al. (2015) PMID: 25999583	FBal0364037	Cas9 mediated mutagenesis
	wls$^{gRNA.U6:3.FP525}$	An ubiquitous promoter drives expression of one Cas9-specific gRNAs targeting *wls*	Port et al. (2015) PMID: 25999583	FBal0364038	Cas9 mediated mutagenesis
	wls$^{gRNA.U6:3.FP522}$	An ubiquitous promoter drives expression of one Cas9-specific gRNAs targeting *wls*	Port et al. (2015) PMID: 25999583	FBal0364039	Cas9 mediated mutagenesis
	wls$^{gRNA.U6:3.FP526}$	An ubiquitous promoter drives expression of one Cas9-specific gRNAs targeting *wls*	Port et al. (2015) PMID: 25999583	FBal0364040	Cas9 mediated mutagenesis

Continued

Table S3. Continued

Target	Name	Features	References	FlyBase ID	Comments
	wls$^{gRNA.U6:3.FP531}$	An ubiquitous promoter drives expression of one Cas9-specific gRNAs targeting *wls*	Port et al. (2015) PMID: 25999583	FBal0364041	Cas9 mediated mutagenesis
	wls$^{gRNA.U6:3.FP534}$	An ubiquitous promoter drives expression of one Cas9-specific gRNAs targeting *wls*	Port et al. (2015) PMID: 25999583	FBal0364042	Cas9 mediated mutagenesis
	wls$^{gRNA.U6:3.FP509}$	An ubiquitous promoter drives expression of one Cas9-specific gRNAs targeting *wls*	Port et al. (2015) PMID: 25999583	FBal0364043	Cas9 mediated mutagenesis
	wls$^{gRNA.U6:3.FP523}$	An ubiquitous promoter drives expression of one Cas9-specific gRNAs targeting *wls*	Port et al. (2015) PMID: 25999583	FBal0364044	Cas9 mediated mutagenesis
	wls$^{gRNA.U6:3.FP520}$	An ubiquitous promoter drives expression of one Cas9-specific gRNAs targeting *wls*	Port et al. (2015) PMID: 25999583	FBal0364045	Cas9 mediated mutagenesis
	wls$^{gRNA.U6:3.FP540}$	An ubiquitous promoter drives expression of one Cas9-specific gRNAs targeting *wls*	Port et al. (2015) PMID: 25999583	FBal0364046	Cas9 mediated mutagenesis
	wls$^{UAS.gRNAx2.pCFD6}$	Expression of two sgRNAs as a single cassette targeting *wls*	Port and Bullock (2016) PMID: 27595403	FBal0351193	Cas9 mediated mutagenesis
	wls$^{gRNA.pCFD3}$	An ubiquitous promoter drives expression of one Cas9-specific gRNAs targeting *wls*	Port and Bullock (2016) PMID: 27595403	FBal0351194	Cas9 mediated mutagenesis
	wls^{HD_CFD01954}	Expression of two sgRNAs targeting coding exons in the 5′ half of the *wls* gene for conditional CRISPR mutagenesis	Port et al. (2020a) PMID: 32053108	FBal0355245	Cas9 mediated mutagenesis. Stock number v342264 VDRC: P{hsFLP}1, y^{1} w^{1118}; P{HD_CFD01954}attP40/ CyO-GFP
	wls$^{KO.attP}$	Amorphic allele, replacement of the *wls* gene coding region with an attP site and a loxP cassette that contains a Disc\RFP$^{mCherry.3xP3}$ marker	Yu et al. (2020a,b) PMID: 32888416	FBal0363564	N.A.
	wls^{ExGFP}	Allele, Evi/wls with a C-terminal GFP insertion in the *wls* locus	Yu et al. (2020a,b) PMID: 32888416	FBal0363565	N.A.
	wls$^{U6:3.3x.LbcrRNA}$	An ubiquitous promoter drives expression of three *wls* targeting LbCas12a-specific crRNA	Port et al. (2020b) PMID: 32843348	FBal0363776	Cas12a mediated mutagenesis
Porcupine (*por*)	por^{15175}	Lethal allele, generated using X-ray	Ferrús et al. (1990) PMID: 2116353	FBal0032471	Stock numbers 4740 BDSC: Ab(1)os^{s}, por^{15175} upd1^{os-s} upd3^{os-s}/ FM6, w^{1}; 86314 BDSC: Ab(1)os^{s}, por^{15175}/FM6, w^{1}; P{neoFRT}82B/TM6C, Sb1 Tb1; 107852 Kyoto: Ab(1)os^{s}, por^{15175} upd1^{os-s} upd3^{os-s}/FM6, w^{1}
	por^{2E}	Lethal allele, generated using ethyl methanesulfonate	Eberl et al. (1992) PMID: 1551578	FBal0032472	N.A.
	por^{G18}	Lethal allele, generated using gamma ray	Eberl et al. (1992) PMID: 1551578	FBal0032473	N.A.
	por^{PB16}	Lethal allele, generated using ethyl methanesulfonate	Perrimon et al. (1989) PMID: 2499512	FBal0032474	Stock number 4768 BDSC: w^{1} ovo^{svb-*} por^{PB16} P{FRTw^{hs}}9-2/FM7a
	por^{i8}	Lethal allele, generated using ethyl methanesulfonate	Eberl et al. (1992) PMID: 1551578	FBal0032475	N.A.
	por$^{hs.PK}$	Transgenic construct containing *por* cDNA	Kadowaki et al. (1996) PMID: 8985181	FBal0056287	N.A.
	por^{+t5}	A wild type genomic rescue fragment	Kadowaki et al. (1996) PMID: 8985181	FBal0056290	N.A.
	por$^{UAS.cTa}$	Transgenic construct containing *por*	Tanaka et al. (2000) PMID: 11821428	FBal0135648	N.A.
	por^{GD3424}	RNAi construct targeting *por* GAL4 system	Dietzl et al. (2007) PMID: 17625558	FBal0210374	N.A.
	por^{GD16931}	RNAi construct targeting *por* GAL4 system	Dietzl et al. (2007) PMID: 17625558	FBal0210375	Stock number v47864 VDRC w^{1118}; P{GD16931}v47864
	por^{KK108587}	RNAi construct targeting *por* GAL4 system	Dietzl et al. (2007) PMID: 17625558	FBal0235670	

Continued

Table S3. Continued

Target	Name	Features	References	FlyBase ID	Comments
					Stock number v100780 VDRC: P{KK108587}VIE-260B
	por^{GLC01423}	RNAi construct targeting *por* GAL4 system	Ni et al. (2011) PMID: 21460824	FBal0281283	Stock number 43236 BDSC: y^{1} sc* v^{1} sev^{21}; P{TRiP.GLC01423}attP2
	por^{B}	Lethal point mutation resulting in premature stop codon (p.R135X)	Yamamoto et al. (2014) PMID: 25259927; Haelterman et al. (2014) PMID: 25258387	FBal0298921	Stock number 57113 BDSC: y^{1} w* por^{B} P{neoFRT}19A/FM7c, P{GAL4-Kr.C}DC1, P{UAS-GFP.S65T}DC5, sn^{+}
	por^{HMC04684}	RNAi construct targeting *por* GAL4 system	Ni et al. (2011) PMID: 21460824	FBal0300023	Stock number 57380 BDSC: y^{1} sc* v^{1} sev^{21}; P{TRiP.HMC04684}attP40
	por$^{fTRG00623.sfGFP-TVPTBF}$	Transgenic construct containing *por* fused to a C-terminal superfolder GFP and other tags	Sarov et al. (2016) PMID: 26896675	FBal0339494	Stock number v318620 VDRC: PBac{fTRG00623. sfGFP-TVPTBF}VK00033
	por$^{TKO.GS01823}$	Expression of one Cas9-specific sgRNAs targeting *por*	Transgenic RNAi Project members, 2017, (personal communication to FlyBase)	FBal0342051	Cas9 mediated mutagenesis. Stock number 79779 BDSC: y^{1} sc* v^{1} sev^{21}; P{TKO.GS01823}attP40
	por$^{TOE.GS01877}$	Expression of two sgRNAs targeting upstream of the transcription start site of *por*	Transgenic RNAi Project members, 2017, (personal communication to FlyBase)	FBal0358255	Transcriptional activation in combination with a nuclease-dead Cas9 fused to a transcriptional activator. Stock number 85789 BDSC: y^{1} sc* v^{1} sev^{21}; P{TOE.GS01877}attP40
	por^{A}	Lethal allele, generated using ethyl methanesulfonate	Graves et al. (2020) PMID: 31767637	FBal0359407	N.A.
	por^{unspecified}	Unspecified *por* allele	Graves et al. (2020) PMID: 31767637	FBal0359408	N.A.
	por^{E}	Lethal allele, generated using ethyl methanesulfonate	Graves et al. (2020) PMID: 31767637	FBal0359409	N.A.

Based on information available at FlyBase, Version: FB2022_01.
BDSC, Bloomington Drosophila Stock Center; Kyoto, Kyoto Stock Center; VDRC, Vienna Drosophila Resource Center; PMID, PubMed identifier.
